# Effect of Toluene Addition in an Electric Arc on Morphology, Surface Modification, and Oxidation Behavior of Carbon Nanohorns and Their Sedimentation in Water

**DOI:** 10.3390/nano11040992

**Published:** 2021-04-13

**Authors:** Kseniya I. Baskakova, Olga V. Sedelnikova, Evgeniy A. Maksimovskiy, Igor P. Asanov, Aida T. Arymbaeva, Lyubov G. Bulusheva, Alexander V. Okotrub

**Affiliations:** Nikolaev Institute of Inorganic Chemistry, Siberian Branch, Russian Academy of Sciences, 630090 Novosibirsk, Russia; baskakova@niic.nsc.ru (K.I.B.); eugene@niic.nsc.ru (E.A.M.); asan@niic.nsc.ru (I.P.A.); arymbaeva@niic.nsc.ru (A.T.A.); bul@niic.nsc.ru (L.G.B.)

**Keywords:** carbon nanohorns, oxidation, specific surface area, aqueous suspension, optical absorption

## Abstract

Carbon nanohorns (CNHs) are attractive for various applications, where a high specific surface area and long dispersion stability in water are important. In the present work, we study these parameters of CNHs prepared by arc evaporation of graphite depending on the conditions of the synthesis and subsequent oxidation in air. It is shown that the addition of toluene in the reactor during the arcing allows obtaining CNHs functionalized with −CH_x_ groups. Heating of CNHs in air at 400 °C leads to substitution of −CH_x_ groups for oxygen-containing groups. Moreover, the CNH endcaps are opened at 500 °C, and as a result, the specific surface area of CNHs increases 4 times. Aqueous suspensions with a concentration of oxidized CNHs of 100 µg/mL are stable for 8 months.

## 1. Introduction

Carbon nanohorns (CNHs) are short tubular structures with conical endcaps, where the wall-constituting carbon atoms are in the *sp*^2^-hybridized states. As a result, they are rigid, chemically inert, and electrically conductive. During the synthesis, the CNHs are combined into spherical particles, which can form large van der Waals associates [1,2,3,4,5]. High pore volume [6,7,8] and specific surface area (SSA) [9,10] make CNHs very promising for nanofluids [11,12], catalysis [13,14], medicine [15,16,17,18], electrochemistry [9,19], electromagnetic [2,5,20] and sensing applications [6,21,22]. In this regard, the improvement of availability of their pores for guest species, the increase of the SSA value, and the enhancement of the stability of their suspensions become very important.

Similarly for carbon nanotubes, oxidation is the most studied method of chemical modification of CNHs [3,15,23,24]. The treatment of CNHs with acids [15,16,25,26] and heating in a presence of oxygen [9,12,22,27,28,29] could etch endcaps of nanohorns or/and open nanowindows in their walls resulting in a significant increase of the SSA value (*ca.* 2.6 and 4 times, respectively [30]). At the same time, the surface modification of CNHs with different oxygen-containing moieties, such as –OH, −COOH, −C=O, −C−O−C− groups [1,3,15,31], is a basic strategy to improve the stability of aqueous suspensions of CNHs. Therefore, both the thermal and acid oxidation of nanohorns allow increasing the SSA and dispersibility of CNHs in solutions simultaneously.

The development of oxygen-containing groups at the CNH surface may occur as a result of the transformation of already attached functional groups. For example, hydrogen atoms in methyl groups can be replaced by O and OH radicals to form CO and COOH groups on the surface of carbon nanotubes [32]. Since the nanotubes and nanohorns have similar structures, this approach can also be suitable for CNHs.

In the present work, we show that −CH_x_ groups present on the CNH surface promote the addition of oxygen-containing groups responsible for the stability of aqueous suspension for at least 8 months. The CH_x_-functionalized CNHs were synthesized directly in an electric arc by evaporating graphitic rods in the presence of different amounts of toluene vapors. The oxidation of the samples was carried out in air at 400 and 500 °C. The samples were characterized by transmission and scanning electron microscopies (TEM and SEM), Raman, infrared (IR), and X-ray photoelectron (XPS) spectroscopies. The SSA values are calculated with the Brunauer–Emmett–Teller (BET) method. The hydrodynamic diameter of the CNH particles after sonication was determined using photon correlation spectroscopy (PCS).

## 2. Materials and Methods

### 2.1. Synthesis of CNHs

The synthesis was performed using a setup for arc evaporation of graphite described in detail elsewhere [2,5,20,33,34]. The arc reactor consisted of a 150 L water-cooled chamber with graphite electrodes placed in its center (a 6 cm diameter hollow cooled anode and a 1.4 cm × 1.4 cm × 20 cm evaporated cathode of ~70 g in weight). All syntheses were carried out until complete evaporation of a cathode. An initial helium pressure was 0.5 bar, direct current was 500 A, and an arc voltage was 50 V. The CNH samples were collected from a metal screen located between a graphite cathode and a cooled chamber walls. The sample prepared by evaporating a graphite rod is denoted as CNH-0. Then, we modified the synthesis process by introducing 2.4 and 8.0 mL of toluene in the reactor chamber during the arcing. The obtained samples were denoted as CNH-2 and CNH-8, respectively. Using more than 8.0 mL of toluene in the synthesis proved to be technically impossible. The addition of toluene increases the pressure in the chamber, thus accelerating the synthesis. The CNH-0 sample was synthesized during 30 min, the manometer connected to the chamber during the arcing showed 1 bar. The addition of 2.4 mL of toluene has not affected the pressure significantly, the synthesis lasted for ~27 min. The synthesis of CNH-8 was finalized during ~9 min, the pressure in the chamber increased up to 1.1 bar.

### 2.2. Oxidation of CNHs

CNH-0, CNH-2, and CNH-8 were thermally oxidized in air in a muffle furnace at 400 and 500 °C for 1 h. The samples were heated from room temperature to the final temperature with a rate of 10 °C/min. The products were denoted as 400-CNH-x and 500-CNH-x (x = 0, 2, 8).

### 2.3. Preparation of CNH Suspensions

CNH suspensions in distilled water (100 µg/mL) were prepared using an ultrasonic disperser (model UZTA-0,15/22-O, U-SONIC Ltd., Biysk, Russia, 70 W, 22 kHz) for 15 min in an ice bath. To determine the effective hydrodynamic diameter of the particles, we studied transparent CNH suspensions (8 µg/mL) obtained by diluting initial suspensions.

### 2.4. Instrumental Methods

The morphology of CNH materials was studied by SEM on a JEOL-6700F (JEOL Ltd., Tokyo, Japan) microscope at an accelerating voltage of 15 kV. TEM images were obtained on a JEOL-2010 microscope (JEOL Ltd., Tokyo, Japan) at an accelerating voltage of 200 kV. The nanohorn sizes were determined by statistical processing of TEM images obtained from four different sites of each sample. Oxygen content was evaluated by energy-dispersive X-ray (EDX) spectroscopy on a Hitachi S-3400N scanning microscope (Hitachi Ltd., Tokyo, Japan) at an accelerating voltage of 20 kV using an INCA Energy 350 spectrometer (Oxford Instruments plc, Abingdon, Oxfordshire, UK).

The SSA of CNHs was calculated by the BET method using the data on low-temperature adsorption of nitrogen (77 K) obtained on a Sorbi MS sorptometer (CJSC «META», Novosibirsk, Russia). The density of initial samples was measured by a pycnometric method in ethanol (Table 1).

The Raman spectra were recorded on a LabRAM HR Evolution HORIBA spectrometer (Horiba, Kyoto, Japan) using a 488 nm excitation.

The functional composition of samples was studied by IR spectroscopy on a FT-801 FTIR spectrometer (Simex Ltd., Novosibirsk, Russia). The spectrum of the KBr pellet and that of the sample were recorded in the region of 400–4000 cm^−1^ in the transmittance mode.

XPS measurements were performed on a Phoibos 150SPECS spectrometer (SpecsGroup, Berlin, Germany) using a monochromatized Al K*α* radiation with the energy of 1486.7 eV. The pass energy of electron energy analyzer was set at 20 eV. The angle between the excitation beam and the entrance of the electron detector was 55°. The base pressure during the measurements was 10^−9^ mBar. The binding energy scale was internally calibrated to the energy 284.5 eV of the C 1s peak. The C 1s and O 1s spectra were fitted using symmetric Gaussian/Lorentzian product function after subtraction of the background signal by Shirley’s method. The C 1s spectral components of *sp*^2^-carbon were described by an asymmetric Doniach-Sunjic function [35].

Effective hydrodynamic diameter of particles was estimated by PCS on a NanoBrook Omni spectrometer (Brookhaven Instruments, Suffolk County, NY, USA) at room temperature. The autocorrelation function was processed by the monomodal analysis. The 640 nm solid-state laser operated at a power of 35 mW, and the scattered photons were detected at an angle of 90° with respect to the radiation source. The acquisition time for one measurement was 10–30 s, the hydrodynamic diameter was determined as the average of 30–60 measurements. The Z-averaged (over intensities) hydrodynamic diameter was calculated for spherical particles using the Stokes–Einstein equation [36]. The calculation parameters included the viscosity of water (1.004 cP) and its refraction index (1.33).

Optical absorption spectra of aqueous CNH suspensions (100 µg/mL) were measured in a 1 × 1 × 4 cm^3^ glass cuvette on an Optizen 220 UV spectrophotometer (K Lab Co., Ltd., Daejeon, Korea) in the region of 400–1100 nm at room temperature.

## 3. Results and Discussion

### 3.1. Morphology of CNHs

**Figure 1:**
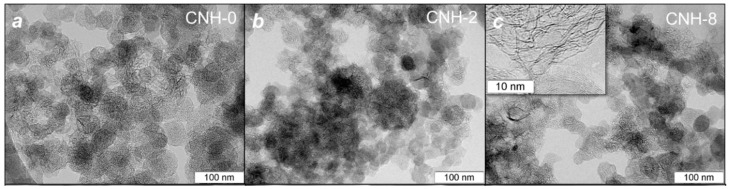
TEM images of samples CNH-0 (**a**), CNH-2 (**b**), and CNH-8 (**c**). Insert in (**c**) shows the image of graphitic-like particles.

**Figure 2:**
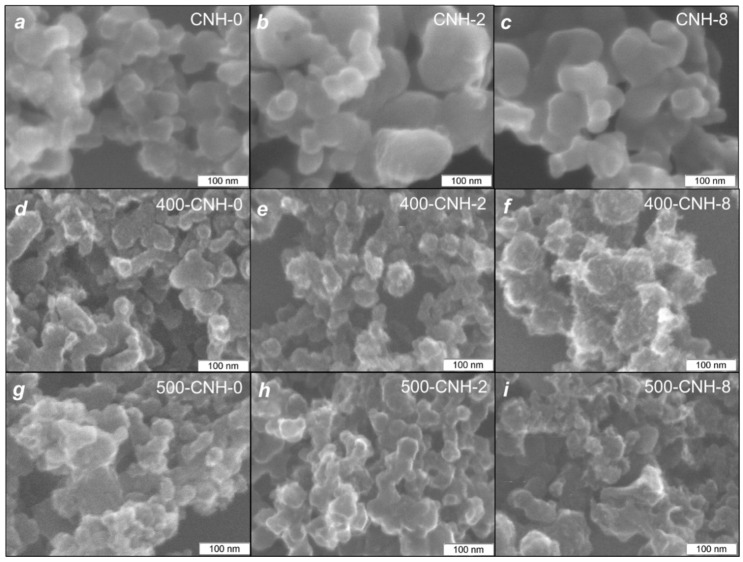
SEM images of initial CNH samples (**a**–**c**) and those oxidized at 400 °C (**d**–**f**) and 500 °C (**g**–**i**).

Figure 1 shows typical TEM images of the samples CNH-0, CNH-2, and CNH-8. The synthesized CNHs can be assigned to the “seed-like” type [37]. Spherical aggregates in the sample CNH-0 consist of individual nanohorns with a length of ~5 nm and a diameter of ~2 nm (for details, see [2,5]). The SEM image of the sample CNH-0 shows 30–50 nm large nanohorn aggregates (Figure 2a). The addition of toluene into the synthesis increased the nanohorn length up to ~10 nm. The samples CNH-2 and CNH-8 mostly contain ~100 nm associates formed by CNH aggregates, whereas individual aggregates are rare (Figure 2b,c).

Moreover, the sample CNH-8 contains graphitic-like particles (insert in Figure 1c). It seems that quick and less uniform carbon arcing due to the injection of 8 mL of toluene provokes the pull-out of the graphite pieces from the evaporated rod. A similar process was observed earlier during arc evaporation of a graphite/melamine mixture [5]. The presence of graphite structures causes the higher density of the sample CNH-8 compared to the values for other CNHs (Table 1).

Further, CNHs were heat treated at 400 and 500 °C in air. The temperature of 400 °C corresponds to the combustion temperature of amorphous carbon [38,39]. Weighing of samples before and after 1 h treatment showed a small weight loss for the samples 400-CNH-x (x = 0, 2, 8; see Table 1) that indicates a high chemical purity of our CNHs from the amorphous carbon. Since single-walled carbon nanotubes have been opened by 500 °C treatment [40], we chose this temperature for the treatment of CNHs. The oxidation at 500 °C decreased the weight of the samples CNH-0, CNH-2, CNH-8 by 19%, 18%, and 8%, respectively (Table 1). According to the SEM study, the treatment of CNHs in air at 400 and 500 °C decreases the size of associates and aggregates (Figure 2d–i). The shape of aggregates differs from sphere-like, indicating partial removal of carbon from the samples.

**Figure 3:**
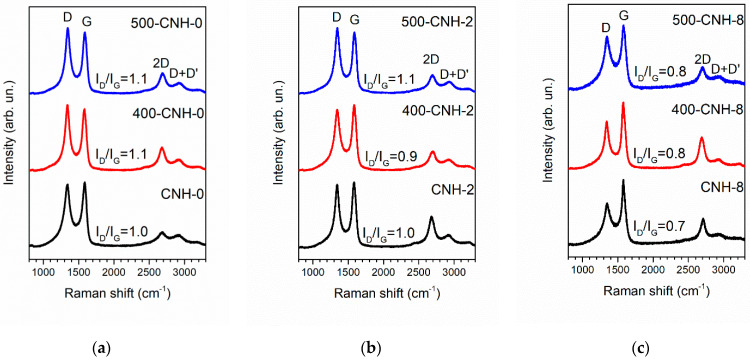
Raman spectra of initial and thermally oxidized samples CNH-0 (**a**), CNH-2 (**b**), and CNH-8 (**c**).

The Raman spectra of our samples are characteristic for *sp*^2^-hybridized carbon materials with defects (Figure 3). The peak at 1580 cm^−1^ is formed by the G mode corresponding to the graphite lattice vibrations. The broad peak at 1350 cm^−1^ corresponds to the D mode, which appears in the presence of defects in the graphite network [41]. The peak 2D at 2700 cm^−1^ is characterized by an overtone of the D band. The weak peak at 2920 cm^−1^ is formed by the overtone of D mode and D’ modes [42,43]. After the treatment of the sample CNH-0 at 400 and 500 °C in air, the ratio between integral intensities of D- and G-bands (*I*_D_/*I*_G_) are almost constant. For 400-CNH-0, the attachment of additional oxygen-containing groups causes an increase of the ratio between the integral intensity of D+D’- and G-bands (*I*_D+D’_/*I*_G_); an increase of 2D band could be due to the enhanced average crystallinity after the removal of an amorphous constituent. The oxidation process was expected to further increase the content of oxygen-containing groups attached to the nanohorns with temperature and to the opening of their endcaps [44]. As a result, the crystallinity of the sample 500-CNH-0 deteriorated, as shown by the decreased intensity of the 2D-band. The spectra of the samples CNH-2 and CNH-8 exhibit a lower value of *I*_D_/*I*_G_ and higher intensity of the 2D band than that of the sample CNH-0. This suggests that the addition of toluene into the carbon arc promotes the formation of nanohorns with more regular graphitic lattice. For 400-CNH-2, the *I*_2D_/*I*_G_ value decreased probably due to the attachment of oxygen groups to the surface. The opening of nanohorn’s endcaps at 500 °C produces dangling bonds, which are attacked by oxygen, increasing the *I*_D_/*I*_G_ value. Among other samples, CNH-8 exhibits a lower *I*_D_/*I*_G_ value.

**Figure 4:**
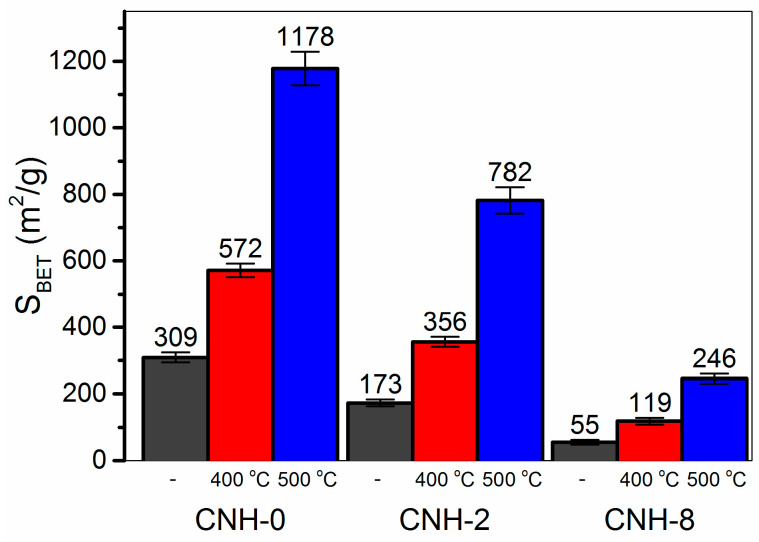
BET specific surface area of initial and thermally oxidized CNHs.

Nitrogen adsorption/desorption isotherms are used to obtain the SSA of porous solids. This approach is also informative for CNHs possessing a hierarchical structure. Figure 4 compares the SSA for initial and oxidized samples. Among the initial CNHs, the sample CNH-0 exhibits the highest surface area of ~309 m^2^/g, which is close to the values reported for carbon nano-onions [45]. This suggests that individual nanohorns have closed endcaps and they are tightly packed in agglomerates. As a result, nitrogen molecules are adsorbed on the external surface of CNH agglomerates, while neither the nanohorns’ cavities nor the inter-horn pores participate in this process. The addition of toluene in the carbon arc significantly decreases the SSA. Taking into account that the samples CNH-2 and CNH-8 mainly contain associates of spherical aggregates, this suggests that inter-aggregate pores in associates are also unavailable for N_2_ adsorption. Moreover, a lower ability to adsorb nitrogen molecules could be due to the decoration nanohorn surface with –CH_x_ groups, as was shown previously [2]. The SSA increases with the treatment temperature for all samples. The oxidation at 400 °C doubles the SSA values of all CNHs. In this case, considering a small weight loss for the samples (Table 1), this treatment partially breakdowns the aggregates without significant influence on individual nanohorns. When temperature increases to 500 °C, the SSA also grows. The maximal value of 1180 m^2^/g is reached for the sample 500-CNH-0 that is comparable with the results reported previously for opened nanohorns (800 m^2^/g [9], 1420 m^2^/g [8], 1450 m^2^/g [44], and 1720 m^2^/g [46]). Moreover, the SSA is close to the theoretical value for opened single-walled carbon nanotubes (1315 m^2^/g [47]). Increased SSA of the oxidized CNHs confirms the assumption that endcaps are opened, making the inner space available for the interaction with nitrogen molecules.

### 3.2. Surface Functionalization of CNHs

**Figure 5:**
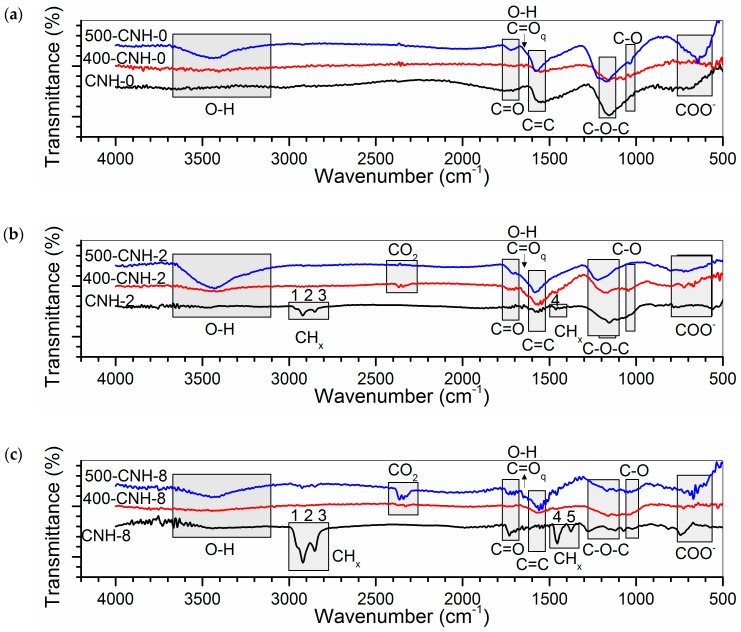
(**a**–**c**) IR spectra of initial and thermally oxidized CNHs. Peaks labeled as 1–5 are assigned to the following vibrations of −CH_x_ groups: asymmetric vibration of CH_3_ fragment (peak 1); asymmetric vibrations of CH_2_ and CH fragments (peak 2); symmetric vibration of CH_2_ fragment (peak 3); asymmetric bending of CH_3_ fragment and the bending of CH_2_ fragment (peak 4); symmetrical bending of CH_3_ fragment (peak 5).

The IR spectra of CNHs are shown in Figure 5a–c. For all initial samples, the bands indicated as C=C stretching vibration of the carbon network are observed at 1550–1560 cm^−1^. Moreover, many bands from functional groups can be seen. Namely, the broad C–O stretching band is located between 1000 and 1300 cm^−1^, the 1730 cm^−1^ band can be assigned to C=O stretching, and the weak O–H stretching band is between 3100 and 3600 cm^−1^. The frequencies of C–O vibrations depend on the details of the coupling to C-C stretching vibrations. In particular, the bands at 1035 and 1150 cm^−1^ could be identified with C–OH and C–O–C stretching vibrations, respectively [48]. The broad absorption band between 600 and 700 cm^−1^ corresponds to the deformation vibrations of COO^−^ in the carboxylate–anion [49]. Additionally, the spectra of samples CNH-2 and CNH-8 exhibit bands 1–5 assigned to CH_x_ vibrations. The pyrolysis of toluene in the arc can be accompanied by the formation of methyl radicals [50,51,52], methylene molecules, and CCH_2_ radicals [53], which interact with the CNH surface to form –CH_3_ and –CH_2_ groups. Asymmetric and symmetric vibrations in CH_3_ cause peaks 1 and 5 in Figure 5b,c, respectively; peak 2 is due to the asymmetric vibration of CH_2_ and CH fragments; peak 3 corresponds to the asymmetric vibration of the CH_2_ fragment; peak 4 is due to the bending in CH_3_ and CH_2_ fragments [54,55]. As the toluene content increases from 2.4 to 8.0 mL, the intensities of these peaks increase significantly. The spectra of oxidized CNHs reveal enhancement of the intensity of the O–H, C–O and C=O stretching bands indicating functionalization of nanohorns with hydroxyl and carboxyl groups and the presence of adsorbed water molecules [16]. The new peaks appeared between 1630 and 1690 cm^−1^ could be related to the oscillation of O–H bands or to the C=O stretching bands of quinone groups. For the latter cause, the frequency depends on the oxygen location in the ring [56]. The peaks of CH_x_ stretching are absent in the spectra of oxidized CNHs, while the absorption bands that appeared at ca. 2322, 2340, and 2362 cm^−1^ are related to CO_2_ molecules in the internal pores of heat-treated CNHs.

**Figure 6:**
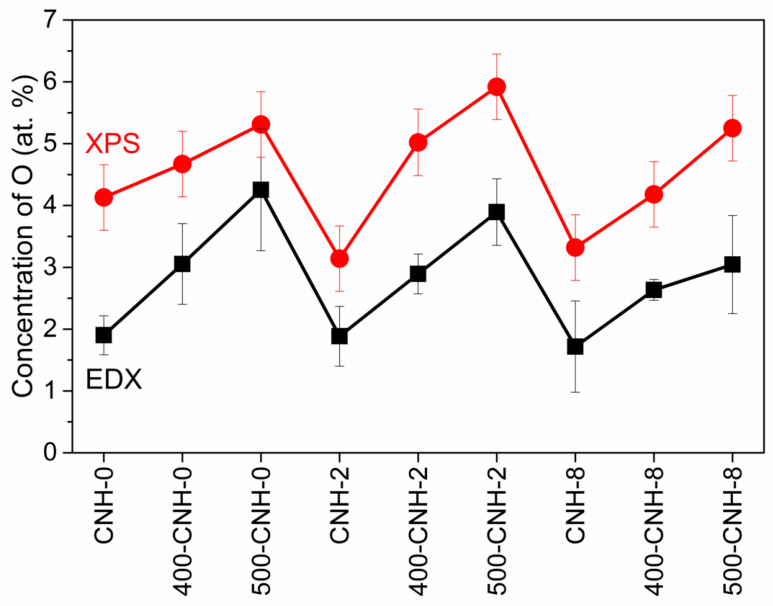
EDX and XPS concentration of oxygen in CNHs.

XPS provides the further probe of the nature of chemical bonds in the CNH samples and transformations of the functional groups as results of toluene addition and heat-treatment in air. The survey XPS spectra of the samples detected carbon and oxygen as dominant elements. The surface concentration of oxygen was estimated to be ca. 4 at.% for the sample CNH-0 and ca. 3 at.% for the samples CNH-2 and CNH-8. The oxygen content remained near the same after 400 °C treatment, while it increased to 5, 6, and 5 at.% for the samples 500-CNH-0, 500-CNH-2, and 500-CNH-8, respectively. The oxygen contents determined by the surface-sensitive XPS method agree well with the data obtained by EDX spectroscopy from a depth of less than 5 µm (Figure 6).

**Figure 7:**
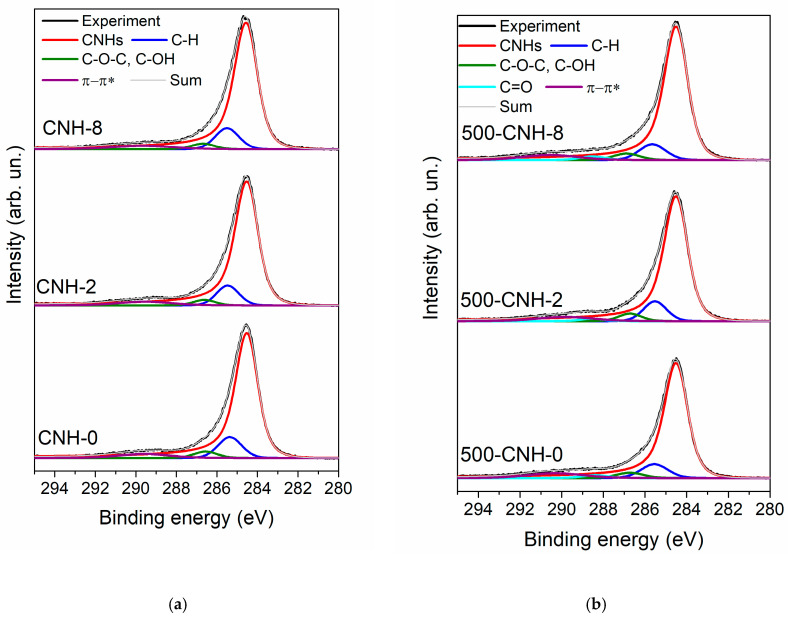

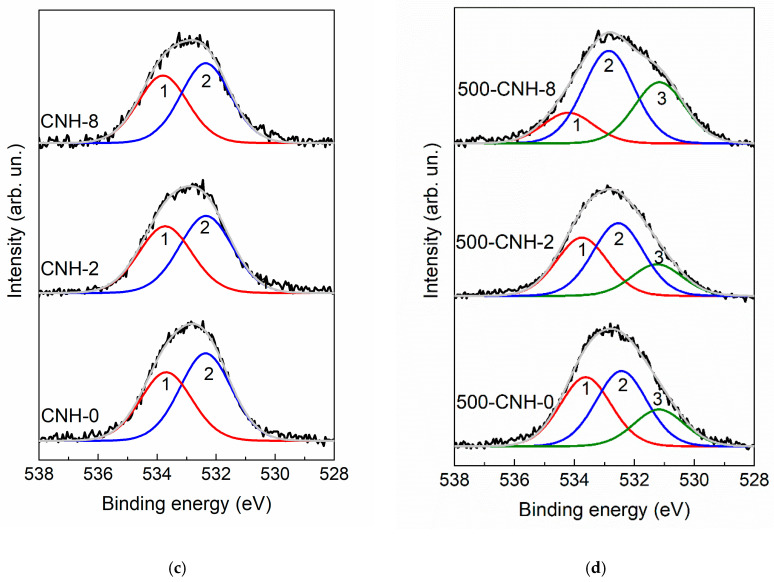
XPS C 1s (**a**,**b**) and O 1s (**c**,**d**)spectra of initial CNHs (**a**,**c**) and those thermally oxidized at 500 °C (**b**,**d**).

Figure 7 shows the XPS C 1s and O 1s spectra of the initial CNHs and those oxidized at a temperature of 500 °C. The XPS C 1s spectra were fitted by four components (Figure 7a,b). A low-energy asymmetric peak with the full width at half maximum (FWHM) of 1.3 eV located at 284.5 eV corresponds to *sp*^2^ carbon atoms [15,35] constituted a well-ordered region of nanohorns. The symmetric peak at 285.3–285.5 eV can be assigned to C–H bonds [57] and *sp*^2^ carbon atoms, which are neighbors to C–O moieties. Attachment of −CH_x_ groups to the graphitic surface requires a radical mechanism as it was shown for methylated single-walled carbon nanotubes [58]. In our case, such molecular C_n_H_m_ radicals are produced directly in the arc by the pyrolysis of toluene. Additionally, the spectral intensity at 285.2–285.5 eV could be associated with C−C bonds between individual nanohorns [59]. Different carbon-oxygen moieties cause the high-binding energy shoulder (C−O and C=O bonds) with an energy of 286–287 eV. The weak band located at ~290.3 eV should be attributed to the shake-up satellite. The energy difference between this peak and the position of main C 1s line (5.8 eV) is significantly lower than that of graphite (6.7 eV), indicating a high corrugation of graphitic layers in nanohorns [60]. After the treatment at 500 °C in air, the amount of hydrocarbon contaminants on the surface of the CNHs is reduced from 9% to 7%. The XPS analysis allows obtaining information from a depth of less than 5 nm, which is approximately equal to the length of an individual nanohorn. The introduction of toluene into the synthesis leads to the formation of large associates over 100 nm in diameter (Figure 2d,g). Thus, most of the −CH_x_ groups located inside the associates are inaccessible for the XPS analysis.

The changes in the XPS O 1s spectra of CNHs occurred after thermal oxidation are similar for all samples against the background of a low concentration of oxygen (Figure 7c,d). The spectra of initial samples have two components located at 533.7 and 532.4 eV (denoted as 1 and 2). Component 1 could be related to H_2_O adsorbed from the air. Component 2 could be assigned to hydroxyl (–OH) and ether (C–O–C) groups [61]. The spectra of samples 500-CNH-0, 500-CNH-2, and 500-CNH-8 contain the additional component 3 located at 531.3 eV, which can be assigned to the C=O bonds in quinone and carboxyl groups [61]. Considering the integrated intensities of peaks 2 and 3, the number of functional oxygen groups on the surface of the sample 500-CNH-8 is significantly higher than that of the other samples.

Based on the analyses of microscopic and spectral data, we propose mechanism of thermal oxidation of CNHs. The treatment of sample CNH-0 at 400 °C in air leads to the functionalization of aggregates’ surface with C–OH and C–O–C groups, which are further oxidized to carbonyl groups. They are attached at defective sites, namely, caps or opened ends of nanohorns. The latter increases the SSA value in agreement with the literature [1,2,8,9,44,46]. The pyrolysis of toluene in carbon arc produces hydrocarbon species. Considering the similarities of XPS and Raman spectra of all initial samples, these additives deposit on nanohorns without significant effect on their defectiveness. According to the calculations given in [32], we suggest that heat-treatment of *sp^2^*-carbon with adsorbed CH_x_-structures in oxidizing atmosphere could transform them into carbonyl groups. Oxidation of modified CNHs groups combines with the functionalization of etched endcaps and sidewall functionalization of nanohorns. This increases the number of C=O groups attached to nanohorns in agglomerates and allows to achieve more uniform coverage of CNH surface with oxygen-containing moieties. This affects the interaction between CNHs and polar solvent molecules. In particular, interacting with water molecules, C=O could be transformed into carboxyl groups. As a result, an aqueous suspension of sample 500-CNH-8 manifests the advanced stability, as discussed in the next section.

### 3.3. Aqueous Suspensions of CNHs

Morphology and surface states of CNHs strongly affect the properties of aqueous suspension, such as dispersion, sedimentation, and extinction coefficient. Table 2 lists hydrodynamic diameters of particles of initial CNHs in water obtained by averaging over distribution function intensities and the number of particles [62]. As can be seen, the light scattering is mainly affected by big particles, whereas the number of small particles in the suspensions is larger. The SEM and TEM images of sample CNH-0 showed 30–50-nm aggregates jointed together to 100-nm associates, while the estimated hydrodynamic diameters are significantly larger. This means the association of CNHs happens in water. For the sample CNH-8, the hydrodynamic diameter increases relatively to the value for the sample CNH-0. This indicates that the interaction between the surfaces of agglomerates modified by −CH_x_ groups becomes stronger, in agreement with conclusions of [2]. Thermal oxidation at 500 °C of the sample CNH-0 changes the particle size slightly probably due to the statistical shift of particle size distribution after combustion of the smaller structures in initial non-modified CNHs. On the other hand, the diameter for the sample CNH-8 was estimated to be approximately halved after thermal oxidation, indicating partial destruction of associates. This agrees with the annealing-induced removal of −CH_x_ groups from the samples synthesized with toluene addition, as was shown by the IR and XPS data.

**Figure 8:**
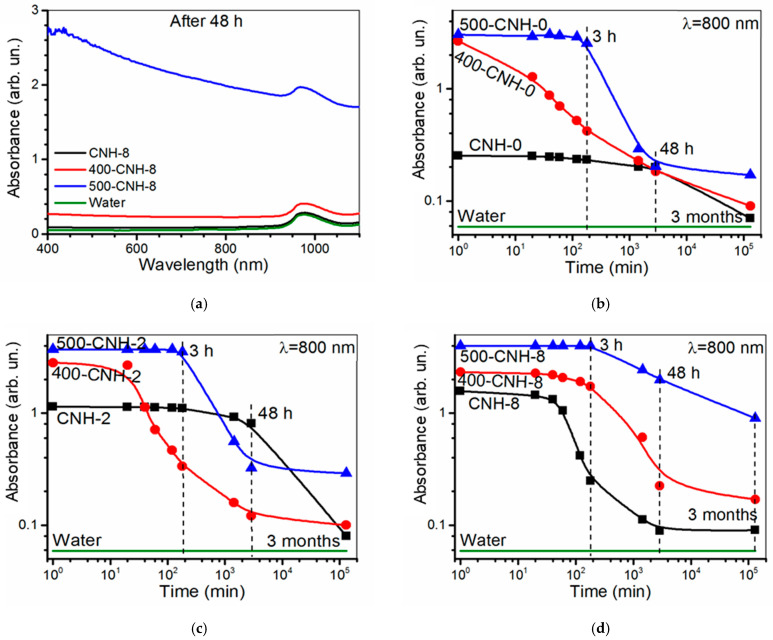

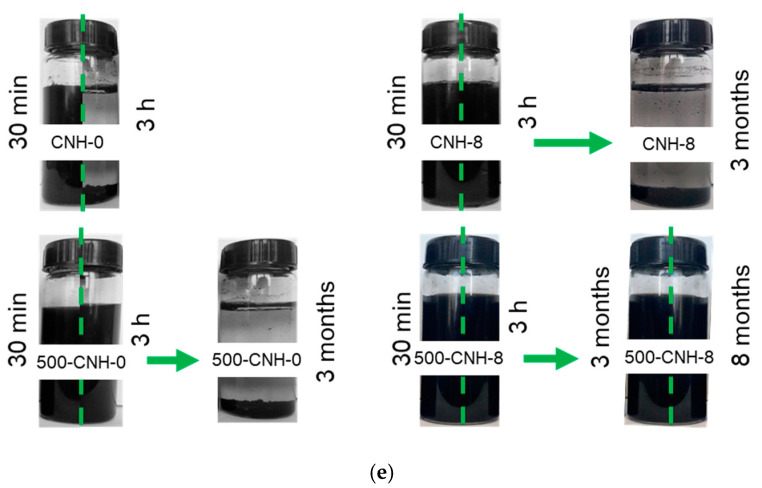
Absorbance of CNH-8, 400-CNH-8, and 500-CNH-8 aqueous suspensions measures in 48 h after sonication (**a**). Absorbance of 800 nm light for CNH aqueous suspensions measured at different time intervals from sonication (**b**–**d**). Sedimentation of suspensions (**e**).

The rate of particle sedimentation and the stability of suspensions were estimated from optical absorption spectra recorded in 1, 20, 40 min, 1, 2, 3, 24, and 48 h, and 3 months after the sonication. A broad absorption peak at 930–1050 nm corresponds to water absorption, while no peaks corresponding to the interaction between water and nanohorns are observed (Figure 8a). The absorption of the aqueous suspensions of heat-treated CNHs increases. This effect can be ascribed to the reduction of particle size with oxidation that agrees with the previous results [15].

The absorbance data acquired at 800 nm are compared in Figure 8b–d. The sample CNH-0 exhibits the hydrophobic behavior over 3 h. For longer intervals, the dispersed particles partially fall down (Figure 8e). This process continuous until the sedimentation of the most particles at the end of the 3rd month. As a result, the spectral intensity is close to that of clean water. Immediately after sonication, the suspensions 400-CNH-0 and 500-CNH-0 are darker than that for CNH-0, however, the oxidized particles sediment during 24 h. It seems that the amount of functional groups on the surface of samples 400-CNH-0 and 500-CNH-0 is not enough to form stable hydrogen bonds with water molecules. The stability of the suspensions CNH-2 and CNH-8 improves compared to that of suspension CNH-0 probably due to the CH_x_-induced enhancement of CNH aggregates interaction with water molecules. The increase of the oxidation temperature allows improving the long-term stability of CH_x_-modified CNHs aqueous suspensions. Despite the sedimentation of some portion of the materials, the suspensions 500-CNH-2 and 500-CNH-8 demonstrate dark coloring even after 3 and 8 months. The hydrophilicity of heat-treated CH_x_-modified CNHs in water could be related to the higher concentration of oxygen-containing functional groups consistent with the oxidation mechanism discussed in the previous section. Namely, the functionalization of etched nanohorns endcaps along with the transformation of −CH_x_ moieties into C=O groups followed by the formation of COOH groups. The obtained dispersibility of the sample 500-CNH-8 is superior to those for CNHs suspensions in water/butanol (stable over several days) [63], and water/ethylene glycol (stable over 6 months for concentrations of CNHs of 5–50 µg/mL) [64], and aqueous suspension of acid-oxidized CNHs (stable over several weeks) [15].

## 4. Conclusions

The morphology of the CNH samples was studied depending on the conditions of arc synthesis and the subsequent heat-treatment in air. The nanohorns are combined into spherical 30–50 nm large aggregates containing numerous cavities inside. The weight loss of the oxidized samples and the SEM data testify that the addition of toluene during arc evaporation of graphite increases contacts between CNHs so that secondary associates are formed. These contacts are most likely provided by −CH_x_ groups present on the CNH surface. The associates of CH_x_-functionalized CNHs are so strong that they persist even after continuous heating at 500 °C in air, and their inner pores do not participate in nitrogen adsorption. The surface area of such CNH samples is relatively small compared to the samples synthesized without toluene additives. In the latter case, the thermal oxidation leads to the opening of nanohorn endcaps, and as a result, the specific surface area increases four times. However, during the thermal oxidation of CNHs, the −CH_x_ groups promote the attachment of oxygen-containing groups responsible for the high dispersibility of CNHs in water. The aqueous suspension with 100 µg/mL concentration of the oxidized CNHs, synthesized using the largest toluene addition, was stable for 8 months at least.

## Figures and Tables

**Table 1:** Pycnometric density and weight loss of carbon nanohorns (CNHs) after heat treatment at 400 and 500 °C in air.

Sample	Density, g/cm^3^	Weight Loss, %	
		400 °C	500 °C
CNH-0	0.26 ± 0.03	7 ± 2	19 ± 2
CNH-2	0.14 ± 0.03	4 ± 2	18 ± 2
CNH-8	0.56 ± 0.03	4 ± 2	8 ± 2

**Table 2:** Hydrodynamic diameters of particles of initial and thermally oxidized CNHs in water obtained by averaging over distribution of function intensities and number of particles.

Sample	Hydrodynamic Diameter, nm	
	Method of Averaging	
	by Distribution of Function Intensities	by Number of Particles
CNH-0	300 ± 20	120 ± 50
500-CNH-0	330 ± 30	140 ± 60
CNH-8	450 ± 30	180 ± 100
500-CNH-8	280 ± 20	110 ± 50
